# Data regarding articles retracted from PubMed indexed dental journals from India

**DOI:** 10.1016/j.dib.2018.03.133

**Published:** 2018-04-01

**Authors:** Thorakkal Shamim

**Affiliations:** Department of Dentistry, Government Taluk Head Quarters Hospital, Malappuram, India

## Abstract

This data aimed to audit the articles retracted from PubMed indexed dental journals from India. The PubMed indexed journals considered are Indian Journal of Dental research, Journal of Indian Society of Periodontology, Contemporary Clinical Dentistry, Journal of Indian Society of Pedodontics and Preventive Dentistry, Journal of Oral and Maxillofacial Pathology and Journal of Indian Prosthodontic Society for type of article, name of dental specialty, topic of individual dental specialties, causes for retraction of article and authorship trend of retracted articles using web-based search. Among PubMed indexed Indian dental journals,the number of retracted articles were as follows: Indian Journal of Dental research (4) followed by Journal of Indian Society of Periodontology (3), Contemporary Clinical Dentistry (3), Journal of Indian Society of Pedodontics and Preventive Dentistry (2), Journal of Oral and Maxillofacial Pathology (2) and Journal of Indian Prosthodontic Society (1). Out of 15 retracted articles from PubMed indexed Indian dental journals, case reports (7) form a major share followed by original articles(6) and review articles (2). Among the dental specialties of retracted articles, oral pathology and microbiology (5) constitute the major share followed by periodontics (4), pedodontics (4), oral medicine and radiology (1) and prosthodontics (1). Duplicate publication (7), plagiarism (5) and authorship dispute (3) are the causes for the retraction of article.

## Specifications Table

**Table:** 

Subject area	*Medicine*
More specific subject area	*Dentistry*
Type of data	*Table*
How data was acquired	*web-based search from the individual dental journal site*
	*1. Indian Journal of Dental Research (45 issues from October–December 2005 to November–December 2014) (*http://www.ijdr.in/backissues.asp*)*
	*2. Contemporary Clinical Dentistry (22 issues from January–March 2010 to October–December 2014) (*http://www.contempclindent.org/backissues.asp*)*
	*3. Journal of Oral and Maxillofacial Pathology (27 issues from January–June 2003 to September–December 2014) (*http://www.jomfp.in/backissues.asp*)*
	*4. Journal of Indian Society of Periodontology (30 issues from January–April 2008 to November–December 2014) (*http://www.jisponline.com/backissues.asp*)*
	*5. Journal of Indian Society of Pedodontics and Preventive Dentistry (76 issues from March 1996 to October–December 2014)*
	*(*http://www.jisppd.com/backissues.asp*)*
	*6. Journal of Indian Prosthodontic Society (20 issues from March 2010 to December 2014) (*http://link.springer.com/journal/volumesAndIssues/13191*)*
Data format	*Descriptive retrospective bibliometric data*
Experimental factors	*Type of article, name of dental specialty, topic of individual dental specialties,causes for retraction of article and authorship trend of retracted articles*
Experimental features	*Bibliometric data*
Data source location	*PubMed indexed individual dental journals site,India*
Data accessibility	*Data are included in the paper*

## Value of the data

These data describe dental sciences related articles retracted from PubMed indexed dental journals from India.

• These data describe baseline data regarding the articles retracted from six PubMed indexed dental journals from India.
• These data describe the number of retracted articles as follows: Indian Journal of Dental research (4) followed by Journal of Indian Society of Periodontology (3), Contemporary Clinical Dentistry (3), Journal of Indian Society of Pedodontics and Preventive Dentistry (2), Journal of Oral and Maxillofacial Pathology (2) and Journal of Indian Prosthodontic Society (1).
• These data describe majority of retracted articles published as case reports (7), original articles (6) and review articles (2).
• These data describe specialty wise majority of retracted articles related to oral pathology and microbiology (5) followed by periodontics (4), pedodontics (4), oral medicine and radiology (1) and prosthodontics (1).
• These data describe duplicate publication (7), plagiarism (5) and authorship dispute (3) as major causes for retraction.
• The data presented in this paper can be used as a guide for conducting similar studies around the globe.

## 1 Data

See Table 1.

**Table 1:** The data regarding articles retracted from PubMed indexed Indian dental journals.

Sl. no.	The data regarding articles retracted from PubMed indexed Indian dental journals	Type of article-dental speciality-topic-causes for retraction-Institution of first author
1	Bansal P, Bansal P. Reconstructive surgery with chin block graft and esthetic rehabilitation of missing anterior tooth. J Indian Soc Periodontol. 2014 Mar; 18(2):263–6.	Case report-periodontics-reconstructive surgery-authorship dispute-Dasmesh Institute of Research and Dental Sciences, Faridkot, Punjab
2	Pedaballi P, Sundaram R, Ramachandran M. Prevalence of gingival enlargement secondary to calcium channel blockers in patients with cardiovascular diseases. J Indian Soc Periodontol. 2012 Jul; 16(3):430–5.	Original article-periodontics-gingival enlargement-plagiarism-RMDCH, Annamalai University, Chidambaram, Tamil Nadu
3	Baghele ON. Buccinator muscle repositioning. J Indian Soc Periodontol. 2012 Jul;16(3):456–60.	Case report-periodontics- anatomical aberrations-duplicate publication-SMBT Dental College and Hospital, Sangamner, Maharashtra
4	Goenka P, Dutta S, Marwah N. Biological approach for management of anterior tooth trauma: triple case report. J Indian Soc Pedod Prev Dent. 2011 Apr–Jun; 29(2):180–6.	Case report-pedodontics-tooth crown fractures- duplicate publication-Government Dental College, Rohtak, Haryana
5	Anand R, Jaiswal JN, Samadi F. Oral pemphigus vulgaris in a pediatric patient. J Indian Soc Pedod Prev Dent. 2010 Jul–Sep; 28(3):200–2	Case report-pedodontics-pemphigus vulgaris-plagiarism-Sardar Patel Post Graduate Institute of Dental and Medical Sciences, Lucknow
6	Rajendran R, Deepthi K, Nooh N, Anil S. α4β1 integrin-dependent cell sorting dictates T-cell recruitment in oral submucous fibrosis. J Oral Maxillofac Pathol. 2011 Sep; 15(3):272–7	Original article-oral pathology and microbiology-oral submucous fibrosis-authorship dispute-College of Dentistry, King Saud University, Riyadh, Saudi Arabia.
7	Bansal S, Shetty S, Bablani D, Kulkarni S, Kumar V, Desai R. Florid osseous dysplasia.J Oral Maxillofac Pathol. 2011 May; 15(2):197–200	Case report-oral pathology and microbiology-florid osseous dysplasia-authorship dispute-Nair Hospital Dental College, Mumbai
8	Koduganti RR, Sandeep N, Guduguntla S, Chandana Gorthi VS. Probiotics and prebiotics in periodontal therapy. Indian J Dent Res. 2011 Mar–Apr; 22(2):324–30.	Review article-periodontics-probiotics-plagiarism-Panineeya Mahavidhyalaya Institute of Dental Sciences and Research Centre, Kamala Nagar, Hyderabad
9	Shetty DC, Urs AB, Manchanda A, Sirohi Y. A color contrast aided density imaging technique to differentiate between dental hard tissues and its relevance. Indian J Dent Res. 2011 Mar–Apr; 22(2):266–9.	Original article-oral Pathology and microbiology-dental hard tissues-duplicate publication-I.T.S Centre for Dental Studies and Research, Muradnagar, Ghaziabad
10	Shetty DC, Urs AB, Ahuja P, Sahu A, Manchanda A, Sirohi Y. Mineralized components and their interpretation in the histogenesis of peripheral ossifying fibroma. Indian J Dent Res. 2011 Jan–Feb; 22(1):56–61.	Original article-oral Pathology and microbiology-peripheral ossifying fibroma- plagiarism-I.T.S Centre for Dental Studies and Research Muradnagar, Ghaziabad
11	Sumanth KN, Boaz K, Shetty NY. Glass embedded in labial mucosa for 20 years.Indian J Dent Res. 2008 Apr–Jun; 19(2):160–1.	Case report-oral medicine and radiology-foreign body-duplicate publication-Manipal College of Dental Sciences, Mangalore
12	Subramaniam P, Prashanth P. Prevalence of early childhood caries in 8–48 month old preschool children of Bangalore city, South India. Contemp Clin Dent. 2012 Jan; 3(1):15–21.	Original article-pedodontics-dental caries-duplicate publication-Oxford Dental College, Hospital and Research Centre, Bangalore
13	Banerjee S, Chakraborty N, Singh R, Gupta T. Full-mouth rehabilitation of a patient with severe attrition using the Hobo twin-stage procedure. Contemp Clin Dent. 2012 Jan; 3(1):103–7.	Original article-pedodontics-full mouth rehabilitation-duplicate publication-Dr. R. Ahmed Dental College, Kolkata
14	Shetty DC, Urs AB, Rai HC, Ahuja N, Manchanda A. Case series on vascular malformation and their review with regard to terminology and categorization. Contemp Clin Dent. 2010 Oct; 1(4):259–62.	Case report-oral pathology and microbiology-vascular malformation-duplicate publication-I.T.S Centre for Dental Studies and Research, Muradnagar, Ghaziabad
15	Sujesh M, Rangarajan V, Ravi Kumar C, Sunil Kumar G. Stem cell mediated tooth regeneration: new vistas in dentistry. J Indian Prosthodont Soc. 2012 Mar; 12(1):1–7.	Review article-prosthodontics-stem cell tooth regeneration- plagiarism-Mamata Dental College and Hospitals, Khammam, Andhra Pradesh

## 2 Materials and methods

There is paucity of information regarding retracted articles in PubMed indexed dental Journals from India. More recently, detailed data of retracted articles in dentistry is published by Nogueira et al. [1]. The data regarding retracted articles in the following dental journals were analyzed for type of article, name of dental specialty, topic of individual dental specialties and causes for retraction of article (last accessed March 2, 2018).

1. Indian Journal of Dental Research (45 issues from October–December 2005 to November–December 2014) (http://www.ijdr.in/backissues.asp).
2. Contemporary Clinical Dentistry (22 issues from January–March 2010 to October–December 2014) (http://www.contempclindent.org/backissues.asp).
3. Journal of Oral and Maxillofacial Pathology (27 issues from January–June 2003 to September–December 2014) (http://www.jomfp.in/backissues.asp).
4. Journal of Indian Society of Periodontology (30 issues from January–April 2008 to November–December 2014) (http://www.jisponline.com/backissues.asp).
5. Journal of Indian Society of Pedodontics and Preventive Dentistry (76 issues from March 1996 to October–December 2014) (http://www.jisppd.com/backissues.asp).
6. Journal of Indian Prosthodontic Society (20 issues from March 2010 to December 2014) (http://link.springer.com/journal/volumesAndIssues/13191).

The data was analyzed using descriptive statistics.
