# Supplementary material for: Data regarding articles retracted from PubMed indexed dental journals from India
Source: Data Brief. 2018 Apr 1;18:1069–72. doi: 10.1016/j.dib.2018.03.133 (PMC5996733; doi:10.1016/j.dib.2018.03.133)
Supplement: Supplementary file 1 — Supplementary material [file mmc1.doc]

**Conflict of interest**

Dear Editor

I did not have any conflict of interest in publishing the research work in the Data in Brief.

Best Regards

Dr. Thorakkal Shamim
